# Infectious diseases associated with pediatric type 1 diabetes mellitus: A narrative review

**DOI:** 10.3389/fendo.2022.966344

**Published:** 2022-08-24

**Authors:** Gianluca Piccolo, Elena Lucia De Rose, Marta Bassi, Flavia Napoli, Nicola Minuto, Mohamad Maghnie, Giuseppa Patti, Giuseppe d’Annunzio

**Affiliations:** ^1^ Department of Neurosciences, Rehabilitation, Ophthalmology, Genetics, Maternal and Child Health, University of Genoa, Genoa, Italy; ^2^ Neuro-oncology Unit, IRCCS Istituto Giannina Gaslini, Genoa, Italy; ^3^ Pediatric Clinic and Endocrinology Unit, IRCCS Istituto Giannina Gaslini, Genoa, Italy; ^4^ Pediatric Clinic and Endocrinology Unit, Regional Center for Pediatric Diabetes, IRCCS Istituto Giannina Gaslini, Genoa, Italy

**Keywords:** diabetes mellitus, children, infections, narrative review, adolescence, immunology

## Abstract

Diabetes mellitus (DM) has been frequently associated with an impaired immune response against infectious agents, making affected patients at risk for more severe disease and sometimes causing worse outcomes. The recent COVID-19 pandemic has seriously affected patients with both diabetes, in particular those carrying comorbidities or with poor glycemic control. As regards pediatric diabetes mellitus, the availability of more accurate and technological tools for glycemic management and the improved markers of metabolic control might mitigate the negative impact of infections. Notably, good metabolic control of diabetes since its diagnosis reduces not only the risk of microangiopathic complications but also of impaired immune response to infectious diseases. Therefore, vaccinations are strongly recommended. Our paper aims to provide the most updated evidence regarding infectious diseases in type 1 pediatric DM.

## 1 Introduction

Type 1 diabetes mellitus (T1DM), arising from a complex interaction between immune, genetic and environmental factors, results from autoimmune-mediated destruction of insulin-producing pancreatic β-cells in genetically predisposed individuals (1). Even if the causative mechanisms are not yet completely defined, T1DM incidence is increasing worldwide (2). Several studies showed that genetic susceptibility alone does not explain the development of the disease and suggested that environmental factors play an important etiopathogenetic role (3). Among environmental factors, the most frequently studied include feeding, infections, gut microbiota, perinatal, and social factors (4, 5). On the other hand, some evidence suggests that lack of exposure in early life to viruses or other infectious agents could increase the risk of T1DM, because of decreased immune stimulation, as supported by the ‘hygiene hypothesis’. It has been hypothesized that in genetically susceptible individuals environmental factors operate by triggering an autoimmune response or overloading the β-cells and promoting apoptosis (4). In T1DM, chronic hyperglycemia and, more recently defined, glycemic variability impairs endothelium function through different mechanisms: oxidative stress, polyol pathway activity, free-radical accumulation, non-enzymatic glycosylation of proteins, free-radical accumulation. All these factors are responsible for the development of various degrees of diabetic microangiopathy, like retinopathy, nephropathy, and peripheral neuropathy (6). Thanks to intensive insulin therapy protocols since diagnosis, reproducible long-lasting and rapid-acting human insulin preparations and advances in technological instruments for T1DM management, clinically evident microangiopathy in children and adolescents is almost rarely encountered. However, subclinical signs of vascular impairment can be detected also in adolescence, and are responsible for increased morbidity and mortality in young adulthood, and for impaired quality of life (7, 8). The natural course of T1DM is sometimes complicated by other autoimmune diseases, in particular celiac disease and thyroid disease. Their prevalence and clinical severity are extremely variable, due to different retrospective studies and data collection (9, 10). Customized insulin therapy, continuous self-monitoring of glucose levels, regular physical activity, adequate dietary habits, and screening for diabetes-related complications and co-morbid conditions are the cornerstone for correct management, long-life expectancy, and satisfactory quality of life (11).Vaccines against most recognized infectious diseases are strongly recommended, especially in pediatric patients (12).

### 1.1 Infections and diabetes mellitus

Even if the role of infections as a trigger of the autoimmune process leading to the clinical onset of T1DM has been extensively studied, few data are available regarding the risk of infections in newly diagnosed and established diabetes. Infectious diseases and T1DM show a bidirectional link: on one hand, poor glycemic control increases the risk of infections, on the other hand, infectious diseases sometimes are the precipitating factor for metabolic decompensation up to diabetic ketoacidosis in both newly-diagnosed and long-lasting patients (13, 14). High blood sugar levels decrease the activity of the immune system and are responsible for changes in tissues, skin, and blood flow, all of which increase the risk of infections (15). If untreated, chronic hyperglycemia impairs leukocyte function and enhances the virulence of several pathogens. In particular, hyperglycemia and/or insulinopenia down-regulate key mediators of host innate humoral and cell-mediated immune response to different pathogens, and reduce the synthesis of pro-inflammatory cytokines (16). As regards cytokines production it has been reported that mononuclear cells and monocytes from T1DM patients secrete less interleukin 1 and interleukin 6. Moreover, chronic hyperglycemia impairs the synthesis of tumor necrosis factor-alpha by T lymphocytes and of IL-10 by myeloid cells (17). Noteworthy, reduced mobilization of polymorphonuclear leukocytes, chemotaxis, and phagocytosis have been observed in the hyperglycemic environment (18). Chronic hyperglycemia impairs host antimicrobial activity by increasing apoptosis and reducing transmigration of polymorphonuclear cells through the endothelium, as well as inhibiting glucose 6 phosphate dehydrogenase (15, 18). Finally, also complement activity is impaired in T1DM patients: lower levels of C4 and subsequent cytokine synthesis and polymorphonuclear dysfunction have been reported (15, 16). Epidemiological studies found that patients with different forms of diabetes mellitus are at increased risk for bacterial complications of pneumococcal infection and for nosocomial bacteriemia, with a mortality rate up to 50% (6). Attention to glycometabolic control reduces morbidity and mortality in T1DM patients, especially within intercurrent illnesses. Strict management of T1DM during intercurrent illnesses is a challenge for pediatric diabetologists (6, 11, 19). If diabetes mellitus itself or degree of metabolic control or underlying immunological dysfunctions influence the susceptibility to infections is debated. Del Roio Liberatore et al. evaluated immunological status in 66 children and adolescents with T1DM and showed only low IgG concentrations and C4B levels related to the degree of glycemic control, without significant impairment of immunological status (20). On the other hand, the impact of infectious diseases on pediatric patients with diabetes mellitus deserves attention. A US retrospective analysis performed on children and adolescents with DM requiring hospitalization for infectious diseases showed a dramatic increase in patients with T1DM (8%), with a longer length of admission (21).

## 2 Methods

A literature search was carried out in PUBMED, Medline, Embase and in Diabetes Societies websites until May 1st, 2022 for articles published between 2000 and 2022. The search terms used included different combinations of: children, adolescents, diabetes mellitus, infections, diseases, vaccines. The bibliographic search was limited to papers published in English and including case reports, original articles, reviews, consensus and metanalyses. The articles were selected on the basis of the title and abstract.

## 3 Narrative review

### 3.1 Respiratory diseases

#### 3.1.1 Tuberculosis

Tuberculosis (TB) is a common comorbidity of diabetes in developing countries and the two diseases share a bidirectional relationship: diabetic people are more prone to contract TB while those with a primary tubercular infection are more likely to have uncompensated diabetes (14). In low and middle-income countries 1.8-45% of inhabitants have TB and 0.1-6% are affected by diabetes mellitus. The risk of TB among people with diabetes mellitus triples that of the healthy population (22) and apparently patient with diabetes mellitus and TB may have an important role in its transmission (23). Notably, patients with T1DM have a higher risk of developing TB when compared to T2DM (24–26).

After primary infection, 90% of individuals with intact immunity control the replication of the bacilli and enter a “latent” phase, asymptomatic, while the remaining 10% develop progressive primary disease. This progression occurs more frequently in patients with poor responses, including unsatisfactory controlled T1DM (as well as HIV infection, chronic kidney failure, or receiving immunosuppressors) (27). Moreover, diabetic patients present an increased risk of TB reactivation, also called post-primary TB, whose symptoms typically begin insidiously along months and affected individuals remain undiagnosed and are potentially infectious for two to three years. These patients can also show more extension cavitation phenomenon (28), or a minimal pleural involvement but more frequently have episodes of haemoptysis (29–31).

TB susceptibility in diabetes mellitus could be explained by the alteration of the function and activation of macrophages, monocytes, and lymphocytes (32, 33); in particular, a reduction in the production of IL-1β in response to *Micobacterium tuberculosis* has been described (34). IL-1β promotes bacterial containment by inducing specific eicosanoids (35) as well as IL-6 production in a paracrine signalling loop. Importantly, this decrease in cytokine signalling is not specific to *Micobacterium tuberculosis*, possibly explaining the higher susceptibility to infection showed by diabetic patients (16, 17). At older ages, a role in this susceptibility is probably played also by pulmonary microangiopathy and renal dysfunction (36, 37).

In a systematic review of 13 observational studies, Jeon et al. (22) found that diabetes was positively associated with TB in all but one study, otherwise the risk of developing an active TB varied significantly from 0 to 8 folds, thus confirming the biologically plausible idea that diabetes mellitus increases on average of 3 folds the risk of TB. A recent meta-analysis by Blanco-Guillot demonstrated convincing molecular epidemiological evidence for TB transmission among diabetes mellitus patients but that there is still not enough evidence to draw conclusions about the propensity for patients with both diabetes mellitus and pulmonary TB to cluster according to the genotype of the infecting bacillus (23). In other words, the epidemiological evidence for the relation between diabetes and tuberculosis is strong, but the molecular and immunological basis is yet to be made clear (34).

TB and diabetes mellitus can be a challenging combination to treat because diabetes can inhibit the host immune response to *Micobacterium tuberculosis* while TB infection as well as some anti-TB drugs, can worsen glycemic control (38), whose efficacy at preventing neuropathy is proven to be more effective T1DM than T2DM (39). Among the issues related to the treatment of TB, the effects of rifampicin on oral antidiabetic agents should be considered; in fact, it could reduce their efficacy and lead to a worse glycemic control (40). Moreover, considering the number of drugs to be used in both conditions, compliance to such a complex polypharmacological treatment needs careful monitoring (41).

Two studies (42) have shown an association between diabetes and multidrug-resistant TB (5-fold more frequent), but contrasting results from other papers (43, 44) make this evidence still inconclusive (45). Intracranial infections (meningitis/encephalitis) caused by TB, as well as *Herpes simplex* type 2 and group B S*treptococcus* can lead to diabetic ketoacidosis (46–48).

Bidirectional screening should be indicated: all patients with TB (both latent and active) should be screened for diabetes mellitus and, on the other hand, all individuals with diabetes coming from nations with a high prevalence of TB infection should be tested with a Mantoux test or Quantiferon detection (44, 49).

#### 3.1.2 Pneumococcus and Influenza

No specific research on the influence of pneumococcal infections and influenza and parainfluenza viruses on children with diabetes is available. Anyway, it is well known that individuals affected by diabetes mellitus are far more likely to be hospitalized because of influenza (50) and that *Streptococcus pneumoniae* represents the first cause of respiratory bacterial infections (15). Therefore, vaccination for these two pathogens is recommended by the American Diabetes Association (51), considering their efficacy in reducing both hospitalization and deaths (52, 53).

#### 3.1.3 COVID-19

The COVID-19 outbreak has recently changed the daily routine of children and adolescents, with a tendency for a sedentary lifestyle and altered dietary habits. Adult patients with T1DM have been included in the high-risk group for COVID-19 infection with high mortality and morbidity rate and priority was given once vaccination was available (54). As regards children with T1DM, in the first year of pandemics evidence was weak. A recent study by Woodruff et al. (55) calculated age-adjusted cumulative population-based rates of severe COVID-19 among 2293 children primarily hospitalized for COVID-19 in the period March 2020 to May 2021 (before vaccination): notably, in the age group 2-17 years diabetes mellitus (both type 1 and 2) affected 88 patients and was associated with increased risk of more severe course (aRR: 1.9; 95% CI: 1.6-2.3).

More T1DM diagnoses in children (56, 57) and increased frequency and severity of Diabetic KetoAcidosis (DKA) at the time of diabetes diagnosis (58) have been reported in European pediatric populations during the pandemic. Using IQVIA health care data from March 2020 to February 2021, the CDC estimated diabetes incidence among patients aged <18 years with diagnosed COVID-19 demonstrating a significantly higher incidence when compared with children without COVID-19 (hazard ratio = 2.66, 95% CI = 1.98-3.56) (59).

In Germany from January 2020 to June 2021, 5,162 children and adolescents with new-onset T1DM were registered, with an observed incidence significantly higher than the expected one (60) and monthly peaks happening about 3 months after each COVID-19 peak. The increased risk for diabetes among children who had COVID-19 highlights the importance of vaccination as an active prevention strategy (19).

### 3.2 Urinary tract infections

Urinary tract infections (UTI) are frequently reported in diabetic patients, mainly related to inadequate glycemic control, diabetic microangiopathy, anatomic abnormalities of the urinary tract and, in girls, recurrent vaginitis (61).The spectrum of UTIs in adults ranges from asymptomatic bacteriuria (ASB) to cystitis and pyelonephritis (sometimes of emphysematous type) to severe urosepsis. The diagnosis of UTI should be suspected if dysuria, urgent urination, sovrapubic pain, or fever with chills occur (62).

Data concerning UTI and ASB in diabetic patients mainly regard adults (63–65) and the possible effect on causing pyelonephritis is unclear. Barnabas et al. found that the prevalence of ASB was higher in 178 children and young adults with T1DM (10.1%) than in matched control subjects (2.6%) and that the spectrum of bacteria in ASB (*Streptococcus agalactiae, Enterococcus* spp.*, Escherichia coli, Klebsiella pneumoniae*) was different from the one typically related to UTI (mainly *Escherichia coli*) (66, 67). As for diabetic women (68), the recommendation of antibiotic therapy in case of ASB in diabetic children remains controversial (69, 70).

*Candida* spp. represents the vast majority of fungal colonization of the urinary tract of diabetic people, causing infection when symptoms or pyuria occur (71).

Emphysematous cystitis (ECy) and pyelonephritis (EP), are characterized by the presence of gas in the bladder wall and in the perinephric tissues or collecting system, respectively. They are more observed in adult women, mainly caused by *Escherichia coli* (72). A single pediatric case of ECy pertains to a young girl aged 15 years admitted to the emergency department for abdominal and back pain and with a history of recurrent urinary tract infections and grade 1 left-sided vesicoureteral reflux: the swab for SARS-CoV-2 resulted positive and an HbA1c of 76 mmol/mol (9.1%) and blood glucose of 200 mg/dL were recorded, consistent with new-onset diabetes mellitus (73).

### 3.3 Gastrointestinal infections

Few data are available regarding surgical illnesses in pediatric patients with chronic diseases. Diagnosis of acute appendicitis in pediatric patients with diabetes mellitus is sometimes difficult, and prompt clinical recognition is a challenge to prevent complications related to a delayed diagnosis. In fact, clinical signs and symptoms (i.e., abdominal pain, vomiting) can be attributed to diabetic ketoacidosis and vice-versa (74). Similarly, delayed gastric emptying due to autonomic neuropathy, even if extremely rare in pediatric patients, could be responsible for abdominal pain (75, 76).

Three reports describe acute appendicitis in pediatric diabetes. In 1988 Latchav reported acute appendicitis in children with diabetes (77). A more recent retrospective examination of the medical records of children with previously diagnosed diabetes mellitus and admitted for acute appendicitis reported clinical characteristics in 18 children, 17 with T1DM. The authors reported a similar presentation as compared to controls, and only fever was less frequent. Perforation was observed in 33% of cases Noteworthy, patients with diabetes showed postoperative severe hyperglycemic crises, suggesting that strict glycometabolic control is mandatory, especially after surgery. No wound complications were reported (78). Finally, a rare case of non-typhoid *Salmonella* infection in a diabetic girl with atypical presentation of acute appendicitis was described (79).

Acalculous cholecystitis has been reported in adults affected by diabetes mellitus, while it is extremely rare in the pediatric age group (80). Emphysematous cholecystitis (EC), which represents less than 1% of cholecystitis, is caused by gas-forming bacteria, (i.e. *Clostridium welchii, Escherichia Coli, Staphylococcus*, and anaerobic *Streptococci*) and its clinical presentation varies from mild abdominal pain to septic shock. EC has been diagnosed in an adolescent with fever, abdominal pain, and vomiting (81). Clinical, laboratory and radiological examinations revealed the amount of gas in the gallbladder wall or in the surrounding tissues were compatible with EC, complicated by pericholecystic fluid collection and secondary acute appendicitis. Gallbladder atony with biliary stasis and small-vessel vasculitis have been considered predisposing factors (82).

Similarly, pyogenic liver abscess (PLA) has been reported in adults, and about 50% of cases affected by diabetes. A fever of unknown origin was diagnosed, and abdominal ultrasound showed a fluid-filled mass in the right hepatic lobe. Percutaneous drainage of the mass revealed an occult pyogenic liver abscess caused by *Klebsiella pneumoniae*, and intravenous antibiotic treatment was followed by recovery. As reported in other cases, unsatisfactory glycemic control is the most important risk factor (83).

The prevalence of fungal infections in human beings has recently increased. *Candida albicans* is the most frequently isolated and proliferates as an opportunistic pathogen as part of intestinal microbiota. Pediatric patients with T1DM showed higher species diversity of the yeast-like fungi, with reduced prevalence of *Candida albicans* as compared to controls, otherwise increased resistance to common antifungal treatment (84). On the other hand, patients with diabetes mellitus are more susceptible to intestinal candidiasis, due to a hyperglycemic environment and to immunological dysfunction. Risk factors are poor glycemic control, prolonged steroids, antibiotics and antifungals use, female gender. Even if vaginal candidiasis has been extensively described in patients with T1DM, few and conflicting data are available on the prevalence of gastrointestinal candidiasis and antifungal susceptibility (85). Kowalewska reported a prevalence of gastrointestinal candidiasis of 75.5% in children and adolescents with T1DM (86), while others found a prevalence ranging from 2.5% to 9.7% (87). On the other hand, oral candidiasis and gingivitis were observed in a cohort of pediatric patients with T1DM and high levels of HbA1c (88). In a cohort of adolescents with T1DM, despite multiple subcutaneous daily injections, serological evidence of hepatitis A, B, C, and E was similar to controls (89).

*Helicobacter pylori* (HP) is a gram-negative bacterium whose frequency has been increasing worldwide, especially among people with low socioeconomic conditions. HP infection affects mainly stomach and several organs of the gastrointestinal tract, otherwise extra gastric manifestations have been described (90). HP has been observed in patients with different age, even in childhood, and if not treated persists over time and is a risk factor for severe complications, including gastritis, gastric and duodenal low-grade lymphomas, hepatic and gastric carcinoma. Moreover, HP infection has been linked to insulin resistance and vascular complications (91). Children with T1DM have been considered at high risk of HP infection and its consequences, even if metabolic control is satisfactory (92). A case-control study in children and adolescents with T1DM reported a higher frequency of HP infection (60%) as compared to controls (40%), positively related to disease duration. Age, insulin requirement, degree of metabolic control, gastrointestinal symptoms frequency were similar between positive or negative patients (93). A longitudinal study aimed to evaluate the reinfection rate of HP in pediatric patients with T1DM showed a higher frequency of HP infection (24%) and reinfection rate (33%) as compared to controls (7% and 4.5%, respectively). Reinfection rate was associated with socioeconomic status and chronological age (94).The eradication of HP in pediatric patients did not improve degree of metabolic control of diabetes (95).

*Ascaris lumbricoides* and *Giardia lamblia* were not frequently reported in a cohort of T1DM patients, nevertheless *Ascaris lumbricoides* has been described as a complicating factor in an adolescent with recurrent DKA (96).

### 3.4 Dermatological infections

It has been reported that up to 30% of patients with diabetes will develop skin abnormalities throughout their lives, frequently related to the time they elapsed from the clinical onset of the disease (97). Among cutaneous complications, infections occur in all age groups, sometimes detectable at the time of clinical diagnosis of both forms of diabetes mellitus.

In children and adolescents with diabetes mellitus severe, recurrent atypical cutaneous infections are frequently described, associated with poor metabolic control and obesity (98, 99). Recently, the widespread use of technological devices for self-monitoring of glucose levels and pumps for insulin administration increased the risk of local dermatological complications (100).

In patients with diabetes, the skin infections more frequently described are fungal and bacterial, while no specific viral strains are involved. On the other hand, an anecdotic report described multiple herpetic whitlows in a child performing self-monitoring of blood glucose (101). Direct contact between damaged skin and secretion containing the virus (i.e. labial herpes) has been considered a causative factor. Pediatric patients with diabetes have a high susceptibility to infections caused by dermatophytes *Candida spp*, including tinea pedis, onychomycosis caused by *Trichophyton rubrum* and *Trichophyton mentagrophytes* (102). Candidiasis of flexures and mucous membranes have been described, in particular vulvovaginitis, balanitis, and angular cheilitis, being reported in up to 5% of patients (103). A prospective study in 32 female children and adolescents with T1DM showed *Candida spp* in 52.5% of cases, with higher frequency as compared to controls (18.2%). *C. albicans* was the predominant species (72.7%), followed by *Candida glabrata* (22.7%), *Candida tropicalis* (2.3%), and *Candida parapsilosis* (2.3%) (104). It is noteworthy that *Candida glabrata* strains are more resistant to fluconazole treatment (105).

As regards bacterial skin infections, *Staphylococcus aureus* has been reported frequently in T1DM, being responsible for infections ranging from skin and soft tissue localization up to more invasive manifestations such as osteomyelitis, septic arthritis, pneumonia, and bacteriemia (106). The role of methicillin-resistant *Staphylococcus aureus* has been studied in adult diabetic foot ulcers, while reports on *Staphylococcus aureus* infection in young patients without foot damage are lacking. Noteworthy, an educational intervention aimed to prevent foot infections in young patients is essential (107). For this purpose, Menne et al. prospectively evaluated all pediatric patients with diabetes mellitus and *Staphylococcus aureus* infection between 2002 and 2010. They identified 47 cases, 43 with skin and soft tissue infections and 4 with osteomyelitis. Female gender, poor metabolic control, obesity, methicillin-resistant strain, and recurrent infections have been reported in the majority of cases (106). Other infections due to *Staphylococcus aureus* are folliculitis and impetigo, while boils and anthrax are rarely encountered (98).

Patients with obesity and diabetes mellitus are affected by intertrigo, characterized by redness in the body folds with secretion and odor, requiring local hygiene, topical antibiotics and in some cases low potency corticosteroids. If not correctly treated, intertrigo can be followed by superinfection with *Candida albicans*, whose treatment includes topical antifungals (98). Other infections are pachyonychia, furunculosis, and anthrax, caused by *Staphylococci*, and erythrasma caused by *Corynebacterium minutissimi*.

The widespread use of technological devices for T1DM management increased the frequency of cutaneous complications. A web-based survey regarding dermatological complications related to pumps or glucose sensors was administered to 139 patients with T1DM from 2 pediatric Italian Centers (108). Skin reaction have been reported in 51.1% of patients, in particular allergic contact dermatitis consisting of vesicles, erythema, edema, and pruritus. It has been hypothesized that repeated taping of the same sites and trauma of repeated insertions are the most important causative factors, together with harmful agents in the devices, like isobornyl acrylate, NN-dimethylacrilamide, and colophonium (108).

The careful examination of the injection sites is mandatory since bacterial colonization of the sensor electrodes has been recently reported (109). The microbiological test performed on sensor electrodes from 31 children with T1DM showed bacterial colonization in 39% of cases. The most frequently encountered strain was methicillin-sensitive coagulase-negativ*e Staphylococcus*, otherwise methicillin-resistant coagulase-negative *Staphylococcus aureus*, *Klebsiella pneumoniae*, *Bacillus sonorensis, Ochrobactrum tritici* have been isolated.

### 3.5 Soft tissues infections

Necrotizing fasciitis (NF) is a severe infection of soft tissues that usually involves the limbs and tacks along fascial planes (110, 111). It has been described in adults with diabetes mellitus as a risk factor for mortality. It is rare in the pediatric age group, and *Varicella-Zoster virus* or *Group A, β-hemolytic streptococcal* infections seem to be predisposing factors. Conwell et al. described 2 adolescents with poorly controlled T1DM who developed polymicrobial NF after different degrees of trauma involving the perineal region (112). *Candida* superinfection and surgical debridement have been reported in one case, and septic shock and suspected osteomyelitis in another. Both patients required prolonged intravenous antibiotic treatment and correction of hypocalcemia complicating the infection. The paucity of cutaneous signs in the early stage of the disease together with intense pain even in absence of tissue damage, otherwise with a history of even mild trauma, should induce suspicion of NF (112).

Another soft tissue complication is iliopsoas abscess (IA). IA is a life-threatening form of extraperitoneal infection involving the psoas and iliac muscles. The classic clinical triad consists of fever, flank pain, and limitation of hip movements (113). Hematological or contiguous anatomical structure spread or local trauma with subsequent hematoma are the causative factors, and *Staphylococcus aureus* is the most frequent microbiological organism isolated in adults. Up to the recent past, IA complicated spine tuberculosis, while more recently predisposing factors are immunological defects, drug abuse, alcoholism, and diabetes mellitus. IA has been observed in adults with diabetes mellitus, while to our knowledge, only 2 case reports described IA in pediatric diabetes. In the majority of cases spinal epidural abscesses were diagnosed (114). Another more recent report described IA in three adolescents with T1DM, poorly controlled in 2/3 cases. All patients showed fever, flank, and/or back pain, requiring prolonged antibiotic treatment; the abscess was diagnosed by Computer Tomography (CT) scan (115).

### 3.6 Rhinocerebral zygomycosis

Zygomycosis is a rare life-threatening opportunistic fungal infection in humans, that can complicate both diabetes mellitus and immunodeficiency syndromes characterized by defects of the cell-mediated immunity (116). In particular, RhinOCerebral Mucormycosis (ROCM) is the most frequent form of mucormycosis in patients with diabetes mellitus (117). The responsible organism is an aerobic saprophytic fungus belonging to the order of *Mucorales*, class of *Zygomycetes* (118). The fungal infection is caused either by inhalation of sporangiospores or *via* direct contamination of skin wounds, and mainly involves the lungs, nasal cavity and paranasal sinuses, gut, and skin (119). Among the mechanisms involved in the higher incidence of mucormycosis in diabetic patients, there are the greater availability of glucose, a lower response of T-cells, and the increased expression of host receptors mediating the invasion of epithelial cells (119). Indeed, in the case of ketoacidosis, the fungal growth is favored by the low pH, due to hyperglycemia as well as higher levels of free iron ions (116). Furthermore, hyperglycemia and the associated metabolic acidosis, are responsible for impaired neutrophil function, neuropathies, and vascular insufficiency, which lead to a diminished resistance to infections and altered tissue response (116).

The prerequisites for the diagnosis of zygomycosis are a high index of suspicion, recognition of host factors, and prompt assessment of clinical manifestations (120). Corzo-Leon et al. proposed an algorithm for the diagnosis of ROMC in diabetic patients: “red flags” include cranial nerve palsy, diplopia, sinus pain, blepharoptosis, proptosis, acute ocular motility changes, internal or external ophthalmoplegia, headache, acute vision loss, periorbital swelling, orbital apex syndrome, and ulcers of the palate (117, 121). Microscopy (both direct and tissue histopathology) and cultures of different specimens represent the gold standard to diagnose zygomycosis. Tissue histopathology shows inflammation in the majority of cases, neutrophilic or granulomatous, sometimes lacking in immunosuppressed patients (120). Imaging (e.g., preoperative contrast-enhanced CT) is a fundamental guide for initial diagnosis, as well as a tool to delimit the infected zone and plan surgical boundaries (121). CT scan will show edematous mucosa, fluid-filled ethmoid sinuses, and destruction of periorbital tissues and bone margins. Notably, bone destruction is generally a late effect occurring after soft-tissues necrosis. Magnetic resonance images (MRI) can also have a role in identifying the intradural and intracranial extent, a cavernous sinus thrombosis, or thrombosis of the cavernous portions of the internal carotid artery. Contrast-enhanced MRI can demonstrate the perineural spread of the infection (117).

The mainstay of mucormycosis treatment is the combination of intravenous antifungal therapy, surgery, and active management of the underlying condition (e.g., decompensated diabetes mellitus). Intravenous liposomal amphotericin B successfully penetrates the central nervous system, thus being used for invasive mucormycosis (122). Posaconazole is a new triazole with a broad antifungal spectrum and its administration seems to be associated with a higher survival rate (116). Di Coste et al. described a case of ROMC pansinusitis in a 14-year-old girl with T1DM; the patient had a 7-day-long history of dental pain, associated with facial swelling, palpebral ecchymosis, and left eye decreased visual acuity, unresponsive to first-line antibiotic therapy. Head MRI showed marked mucosal thickening of the left maxillary sinus extended to the sphenoid, ethmoid, and frontal sinus. Inflammatory tissue was excided through an endoscopic sinus surgery and cultures revealed growth of *Zygomycetes*. Due to the severe impairment and extended infection, intravenous amphotericin B was started but shortly switched to posaconazole because of severe side effects (hyperglycemia, marked hypothermia, and profuse sweating). A very slow improvement was observed, with poor glycemic control. Unfortunately, the patient ultimately developed blindness in her left eye (116). In conclusion, given the rapid course of ROCM and the high mortality risk if the microorganism overcomes the skull, every pediatric patient with T1DM and signs or symptoms belonging to the abovementioned “red flags” should be a candidate for prompt imaging evaluation and nasal endoscopy, in order to rule out mucormycosis and start antifungal therapy.

### 3.7 Oral infections

Infections of the oral cavity represent a heterogeneous group of infectious diseases that can be caused by a wide range of microorganisms. Children with T1DM are at higher risk of developing oral infections than the healthy pediatric population, this being a consequence of reduced salivary flow, modified salivary composition (particularly in decompensated diabetes), and a change in the oral bacterial flora (88, 123). A larger secretion of inflammatory mediators, the impaired immune cell function, the glycation of proteins, and the altered wound healing are all factors potentially contributing to more frequent and sometimes also more severe oral infections in T1DM (123). In detail, the impaired immune cell function consists of defective chemotaxis and reduced phagocytosis; diabetic patients with severe infection have depressed PMN chemotaxis compared to those with mild infection or non-diabetic subjects with severe or mild infection (123). Moreover, the synthesis, maturation, and homeostasis of collagen seem to be affected by glucose levels, undergoing nonenzymatic glycation which results in the irreversible formation of altered proteins known as Advanced Glycation End-products (AGEs). Notably, high levels of AGEs determine enhanced oxidative stress at the level of gingival tissues. TNF-α also has a crucial role in case of infections in patients with TIDM, stimulating fibroblasts to synthesize matrix-degrading enzymes and suppressing insulin peripheral effect by both phosphorylations of a serine residue of the Insulin Receptor Substrate 1 (IRS-I) and reduction of the mRNA expression of IRS-I and GLUT-4 (124–126).

Ueta et al. demonstrated that diabetes mellitus is a predisposing condition for odontogenic infections and oral candidiasis; moreover, diabetes-complicated infections are more severe, with higher C-reactive protein and erythrocyte sedimentation rate levels (127). Gomez-Diaz et al. evaluated the association between carotid Intima-Media Thickness (cIMT), buccodental status, and glycemic control in children with T1DM and found that those with HbA1c > 69 mmol/mol (8.5%) had greater frequency of caries, gingivitis, and enamel demineralization, and were positive for *Streptococcus mutans* and *Candida albicans*; moreover, in these patients cIMT increased and vessel compliance decreased compared to those with HbA1c <53 mmol/mol (7.0%; p < 0.05) (128). Safia et al. compared salivary samples from diabetic patients with healthy controls and showed that diabetics had a higher candidal carriage rate compared to controls; in particular, *Candida albicans* was the most frequently isolated species, but diabetics patients had a much wider variety of species compared with controls (129).

*Candida albicans* is among the most frequent causes of oral cavity infections in children with T1DM, being responsible for various clinical forms including maxillofacial involvement, pseudomembranous form (classic onset, with xerostomia), rhomboid glossitis, perioral dermatitis, and angular cheilitis (125). Arslan et al. showed significant differences in terms of isolated *Candida* frequency between healthy subjects and diabetic patients, but no difference between the groups in terms of virulence (130). Cytologic preparation is essential for diagnosis, being the morphologic forms of *Candida spp* the most significant finding (with the bacterial load which normally colonizes the mouth being an interesting, but minor element). The azole class of antifungal medications (e.g., itraconazole, clotrimazole, fluconazole) exerts its action by preferentially inhibiting fungal cytochrome P450, an essential element to continue ergosterol production and let the microorganisms grow (129).

Olczak-Kowalczyk et al. compared a group of children with T1DM and no other chronic disease and a group of healthy children for oral fungal infection and its course. *Candida spp* often occurred in healthy patients, but oral candidiasis was found only in the diabetes groups (11.4%). Gingivitis occurred more frequently in patients with diabetes and its severity was correlated with higher glycemia and glycated haemoglobin > 64 mmol/mol (8%) (88). In conclusion, oral cavity infections and T1DM are bi-directionally associated: the inadequate control of glucose levels may potentiate the severity of plaque-related gingivitis.

## 4 Conclusions

We evaluated the different infectious diseases associated with pediatric diabetes mellitus. It is noteworthy that, despite the improved management of the disease, infections still represent a severe risk factor, especially in cases of unsatisfactory control. An unusual initial clinical presentation or symptoms attributed to diabetes itself can delay diagnosis and a more severe course of infection can negatively affect the outcome. Good metabolic control and careful clinical evaluation are the cornerstones to prevent infections, together with adherence to the recommended vaccination program. Pediatric diabetologists should be aware of the risk connected with infectious diseases, and patients should be adequately taught about the “sick day management” to prevent DKA.

## Author contributions

Conceptualization: GPi and Gd’A. Methodology: GPi, NM, FN, GPa, and Gd’A. Writing—original draft preparation: GPi, ER, MB, and Gd’A. Writing—review and editing: GPi and Gd’A. Supervision: GPa, MM, and Gd’A. All authors have read and agreed to the published version of the manuscript. All authors contributed to the article and approved the submitted version.

## Conflict of interest

The authors declare that the research was conducted in the absence of any commercial or financial relationships that could be construed as a potential conflict of interest.

## Publisher’s note

All claims expressed in this article are solely those of the authors and do not necessarily represent those of their affiliated organizations, or those of the publisher, the editors and the reviewers. Any product that may be evaluated in this article, or claim that may be made by its manufacturer, is not guaranteed or endorsed by the publisher.
